# A Cab Ride

**DOI:** 10.4269/ajtmh.19-0300

**Published:** 2019-09

**Authors:** Seth D. Judson

**Affiliations:** University of Washington, Seattle, Washington

It is midnight at Logan International Airport as I walk outside into the brisk Boston air. I wait in line as cabs swing into formation. A driver wearing dark glasses and a knit beanie beckons me over. Hurriedly, we speed off into the December night. The shadowy face of the driver is intermittently illuminated by fluorescent lights dotting the streets. I notice that he is chewing on a stick. It reminds me of a stick that my soccer coach from Ghana used to chew during practice, so I ask him about it. “They have a different name for it in Ghana,” he replies. “This one is harder…I am from Senegal, where are you from?” I tell him that I am visiting from Montana to interview for medical school. “What type of medicine?” he asks me. I explain that I do research with the National Institutes of Health in Montana on Ebola virus and am interested in emerging infectious diseases. The year is 2014, and we are in the midst of the West African Ebola virus disease epidemic, so this response tends to either end conversation, making people shift uncomfortably in their seats, or spark interest. He is interested.

“Why is this outbreak occurring?” he asks. I share with him about the virus and how we think that bats carry it in nature and that it rarely spills over into humans, but when it does, it can spread rapidly in areas with limited public health infrastructure and sanitation. “I am Muslim,” he responds. “My prophet says that you should always be clean. We do not eat bats or monkeys like they do,” he says shaking his head. I ask him more about his culture and family in Senegal. “I fear for them…that someone could bring it there,” he replies, staring off into the darkness.

His words remind me of a recent topic of conversation back at the laboratory in Montana. A case of Ebola virus disease had recently been detected in Mali. Members of the research team who work in Mali were concerned that it could spread quickly there and in other Muslim-majority countries because of an Islamic tradition of open borders. “I have heard that part of Islam is to never close your borders to your neighbors, to always provide help…which may make it harder to stop the spread of disease…is that true?” I ask.

“No!” he exclaims, removing the stick from his mouth. “My prophet says that you close your borders in times of disease!” Sitting in the back of the cab, I think about those conversations back at the laboratory. I also think about what he said about the behaviors of those not belonging to his religion. We both had made assumptions. But if we had not spoken, perhaps some of those assumptions would have persisted. Perhaps some of the cultural nuances he shared could also be used to improve our understanding about Ebola virus.

After a while he asks me, “So why do you want to help?”

“I…I feel like it is my duty, since I have learned so much about Ebola…I have been fortunate,” I reply.

“It is your duty,” he agrees, as we pull up to my stop.

A year and a half later, I am in the back of a cab in Yaoundé, Cameroon, weaving through crowded streets. Dodging motorcyclists and fruit vendors, we pull off into a dirt parking lot. As I climb out of the car, I am greeted by a merchant who is cooking safou, a purple oblong fruit, on a charcoal pit. I purchase the safou and he hands me the charred fruit, his own fingers blackened by the coals. As I peel back the skin and try the buttery flesh, I think about my upcoming meeting.

I am here to discuss risk maps of Ebola and other emerging viruses with researchers and policymakers as part of my medical school summer research. Back during the Ebola outbreak, we had made predictive maps for Ebola virus, using spillover events to identify habitats where the virus might emerge. My goal is to figure out whether these maps are useful to national policymakers and how they can be improved with local knowledge. Although I have only arrived in Cameroon, I have already met with various ministries and research institutions to discuss the maps. Our conversations had started with me explaining the research and then diving into a series of standardized questions. These meetings had felt stifled and one-sided, so as I chew on the safou, I pick a new approach.

After my colleague and I introduce ourselves to the next group of national experts, I say, “We are here to learn from you. You are the experts about Cameroon, and we want to figure out how our research can be more helpful.” I notice their faces relax and the corners of their mouths turn into slight smiles. They share how they are tired of people coming here and telling them how to do things in their country. They further open up to me, eager to share ideas and feedback that we have not yet heard.

As I leave the meeting and head off to meet with other policymakers, I think about our dialog. I think about the cab driver from Senegal. I think about assumptions, those that I carried with me in the back of the cab in Boston and those that I brought with me to Cameroon. The field of tropical medicine provides the opportunity to have conversations that clarify assumptions, allowing people to work together to solve both global and local issues. However, these conversations will not happen without humility, respect, and open curiosity for one another.

